# Invisible walls? Stigma-related perceptions are associated with reduced help-seeking intentions for disordered eating in men

**DOI:** 10.1186/s40337-024-01152-3

**Published:** 2024-12-05

**Authors:** Martin S. Lehe, Georg Halbeisen, Sabine Steins-Loeber, Georgios Paslakis

**Affiliations:** 1 University Clinic for Psychosomatic Medicine and Psychotherapy, Medical Faculty, Campus East-Westphalia, Ruhr-University Bochum, Virchowstr. 65, 32312 Lübbecke, Germany; 2 Department of Clinical Psychology and Psychotherapy, Otto-Friedrich-University of Bamberg, Markusplatz 3, 96047 Bamberg, Germany

**Keywords:** Eating disorders, Male, Men, Stigma, Gender, Treatment barrier, Access to care, Help seeking

## Abstract

**Background:**

Eating disorders (EDs) are increasingly prevalent in men, but men remain underrepresented across many ED-specific treatment settings. Based on the idea that persistent stereotypes, prejudice and discrimination, i.e., stigma against men with EDs, could impede help-seeking behaviors, the present study investigated whether stigma-related perceptions in men are associated with reduced help-seeking intentions for a broad range of disordered eating symptoms.

**Methods:**

*N* = 132 adult men participated in a cross-sectional online survey and completed questionnaires on ED psychopathology, muscle dysmorphia, orthorexic eating, stigma-related perceptions of EDs in men, and help-seeking intentions.

**Results:**

Moderator analyses showed that higher stigma-related perceptions were associated with reduced help-seeking intentions in response to increased ED symptom severity. However, this was only the case for traditionally “feminized” ED symptoms (related to thin-body ideals), but not for help-seeking with regard to muscularity-oriented, orthorexic, or avoidant/restrictive disordered eating.

**Conclusions:**

Stigma may reduce help-seeking intentions with regard to “feminized” ED symptoms. The present findings suggest that perceptions of EDs as “women’s diseases” were associated with reduced help-seeking in men. Stigma towards men with EDs could thus be a possible barrier to help-seeking in men, highlighting the relevance of stigma-reducing interventions in clinical and community settings.

## Abstract (highlights)

Eating disorders are associated with many burdens for those affected, their relatives, and the healthcare system. In recent years, there has been a significant increase in the incidence of eating disorders—particularly among men. Nevertheless, data shows that men are less likely than women to receive adequate treatment. Drawing on reports of individuals with lived experience, several barriers to treatment are commonly discussed in the literature. One of these is stigma, commonly present in men with eating disorders. This study empirically supports the notion that stigma-related perceptions may be a barrier to seeking treatment for men with disordered eating. This may help to develop interventions on different levels to break down barriers to treatment for men.

## Background

Eating disorders (EDs) are severe psychiatric conditions characterized by body image concerns, eating disturbances, and weight-control behaviors that impose substantial levels of individual [1, 2], caregiver [3, 4], and economic burden [5]. Although considered one of “the most gendered of all disorders” [6], EDs affect a socially diverse range of individuals [7, 8], including adult men. In fact, ED prevalence in men increased by 22% over the past three decades compared to a 12% increase in women [9]. Women remain in the majority, but current lifetime estimates of 2.2% ED prevalence in men and 8.4% in women suggest that men could account for every fourth to fifth clinical case [8, 10].

However, despite seminal case reports of men with EDs [11–13], men remain underrepresented across many ED treatment settings [14]. This may be because men’s symptoms may go unnoticed due to gender differences in body image and presentation of disordered eating [15–17]. For example, disordered eating in men has been associated with orthorexia symptoms, exercise dependence, the drive for muscularity, and muscle dysmorphia [18, 19]. In some settings, lesser-known ED diagnoses may also be more common in boys and men (e.g., avoidant/restrictive food intake disorder, ARFID, in pediatric gastroenterology [20]. Still, one of the most prominent reasons for low ED treatment rates in men may be persistent stereotypes, prejudice, and discrimination, that is, stigma against men with EDs and subsequently delayed or thwarted help-seeking behaviors [21].

Adult men presumably delay seeking help for health-related issues, particularly those pertaining to mental health, due to the conflict between help-seeking behavior and ideals of masculinity and traditional gender roles [22, 23]. Traditional gender roles require men to be strong and self-reliant, and to avoid showing weakness and vulnerability [24]. These gender roles are socially reinforced, such that individuals experience reward for adhering and social backlash, i.e., social and economic penalties, for not adhering to societal expectations [25], incentivizing inaction regarding the disclosure of problems and seeking help. Furthermore, manhood may be perceived as a status that is precarious and potentially lost by displaying “feminine” behaviors or behaviors that fail to demonstrate masculinity [26]. Thus, seeking help could not only conflict with masculinity ideals and traditional gender roles, but actively threaten a man’s perceived social status [27]. Consequently, seeking help for having disordered eating, a “feminized” mental health problem that violates gender ideology, appears particularly problematic and might face significant social and individual barriers.

Indeed, two recent systematic reviews suggest that men with disordered eating encounter unique challenges in accessing care. Richardson and Paslakis [28] summarized qualitative studies on men’s lived experiences, revealing, for example, men with EDs having trouble finding specialized treatments, physicians not taking symptoms in men with EDs seriously, and men delaying help-seeking due to the internalized notion that EDs are a “women’s disease”. Including quantitative studies and focusing specifically on barriers to help-seeking in men, Bomben et al. [29] similarly concluded that perceiving services as “feminized” and EDs as a “female illness” have adverse effects on men. Specifically, five cross-sectional surveys showed that men and women were equally likely to recognize ED symptoms [30] but that men with a diagnosed [31] or self-suspected ED [32] expected more negative consequences from seeking psychological help in general compared to women. Such generalized negative expectations further emerged as a stronger predictor of lower help-seeking intentions in men with disordered eating compared to women [33, 34], which Bomben et al. [29] attributed to a feminized construction of EDs actively thwarting men’s chances of recovery. After all, admitting one’s symptoms [35] and then intending to seek help [36] are initial steps and significant predictors for recovery and ED treatment outcomes.

While the overall findings are consistent with a critical association between delayed help-seeking and stigma-related perceptions of EDs in men (e.g., “EDs are a women’s disease.”; “There aren’t any services for men.”; “Physicians will not take men’s symptoms seriously.”), it is important to note that the previous studies rarely tested the impeding effect of these stigma-related perceptions directly. The cited studies almost exclusively measured men’s perceptions towards seeking psychological help in general. The pronounced rates in men [31] and associations with disordered eating [32] or general help-seeking intentions [33] thus only indirectly support the notion that it is stigma-related perceptions towards men with ED that are associated with reduced help-seeking intentions. None of the cited studies actually measured stigma-related perceptions of EDs in men, which could be distinct from generalized negative expectations of seeking help [37]. In addition, previous studies with men mainly included “female-centric” disordered eating assessment (e.g., the Eating Disorder Examination-Questionnaire; [38, 39]) and did not consider stereotypically “male” body image concerns and disordered eating presentation [6, 40]. It, therefore, remains an open question whether reduced help-seeking intentions for other disordered eating symptoms would be similarly associated with stigma-related perceptions of EDs in men or if the effect, as is currently assumed [29], specifically concerns “feminized” disordered eating symptoms.

To address the open questions and extend upon previous knowledge on how stigmatization could affect men [41], we investigated the effect of stigma-related perceptions of EDs in men on men’s help-seeking intentions for disordered eating symptoms. Specifically, we collected data on men’s disordered eating symptoms, their stigma-related perceptions of EDs in men, and their help-seeking intentions in a cross-sectional survey. We generally assumed increased help-seeking intentions in individuals admitting to more disordered eating symptoms [35]. However, based on the idea that a feminized construction of EDs is associated with reduced help-seeking behaviors in men [28, 29], we further predicted that more pronounced stigma-related perceptions of EDs in men would moderate, i.e., reduce the relation between symptom severity and help-seeking intentions. Because we collected data on a broader spectrum of disordered eating behaviors, we further explored whether this association specifically occurred with “feminized” disordered eating symptoms (i.e., those related to thin body ideals and weight control behaviors) or also disordered eating symptoms potentially more relevant—and less stigmatized—in men (e.g., muscularity concerns, or ARFID [6, 15]).

## Materials and methods

### Participants and design

The present study included data of *N* = 132 adult men (*M*_age_ = 33.4, *SD*_age_ = 12.4, age range: 18–68 years) as a subset of a cross-sectional online survey [42]. The initial study comprised a sample of 158 participants in total, from whom 146 were men meeting the inclusion criteria. Participants were recruited from the local university campus, in online social networks, and among acquaintances between May 2022 and June 2023. For the present analysis, we included the subsample of adult men who answered ‘no’ to having ever received a diagnosis for an eating disorder, given that we were interested in predicting help-seeking intentions unaltered by prior ED-related help-seeking experiences. An a priori power analysis was conducted using G*Power version 3.1.9.7 [43] for sample size estimation. This was based on the assumption of a small-to-medium-sized effect (ρ = 0.10) according to Cohen’s criteria [44], which had been observed in previous research [45]. With a significance criterion of α = 0.05 and power = 0.80, the minimum sample size needed with this effect size was *N* = 100 for moderation analyses with two interaction effects. Thus, the obtained sample size was adequate to test the study hypotheses.

The survey was approved by the Ethics Committee of the Ruhr-University Bochum’s Medical Faculty at Campus East-Westphalia (AZ 2022–910, April 21st, 2022), prospectively registered at https://aspredicted.org/5T3_NH5, and conducted in accordance with the Declaration of Helsinki. All participants gave informed consent. Participants received no compensation. We report all measures and exclusions. Data and materials can be obtained from the corresponding author upon request.

### Measures and procedure

The online survey was hosted on our webserver and implemented using jsPsych [46]. The study was advertised as a survey on improving the detection of disordered eating in men. Upon accessing the study’s website, the study information and consent forms were presented. We explained that the survey included the assessment of disordered eating and other symptoms in men and that participants could receive feedback on their risk for an eating disorder. Participants first provided sociodemographic information (including age, weight, height, regular medications, gender, sexual orientation, German language proficiency, migration background, years of education, marital status, living circumstances, ED history and treatment, and previous study participation). The latter question served as a quality check to exclude records from repeated participation. Participants next completed an ED risk assessment (EAT-8 [47]; for further details, see [42]), and, in randomized order, disordered eating symptoms, help-seeking intentions, and stigma-related perceptions of EDs in men. The survey took between 15 to 20 min to complete.

#### Disordered eating symptoms

Similar to previous studies [31, 32], we used the Eating Disorder Examination-Questionnaire (EDE-Q; [48, 49]) to assess cognitive and behavioral ED symptoms within the preceding 28 days. The EDE-Q encompasses four subscales, namely “(Dietary) Restraint”, “Eating Concern”, “Shape Concern”, and “Weight Concern” utilizing 22 attitudinal items rated on a 7-point scale (ranging from 0, *never*, to 6, *every day*). Six additional open-ended items assessed overeating episodes, binge episodes, binge days, and purging behaviors but were not used in scale construction. Since earlier investigations did not endorse the proposed factor structure of the EDE-Q in men [39], we opted not to consider the subscale scores. Instead, we used the global mean score across attitudinal items (Cronbach’s α = 0.94, McDonald's ω = 0.94).

Extending upon previous studies, we also assessed additional disordered eating and associated symptoms. The Muscle Dysmorphic Disorder Inventory (MDDI; [50]) comprised 13 items, rated on a 5-point scale (from 1, *never*, to 5, *always*), assessing disturbances and behaviors associated with muscularity-related body image (e.g., the belief that one’s body is too skinny, hating one's body, and experiencing depressed mood when not engaging in exercise; Cronbach’s α = 0.82, McDonald's ω = 0.76). The Duesseldorf Orthorexia Scale (DOS; [51]) assessed the preoccupation with healthy eating and related behaviors in the past week using ten statements (e.g., “I prioritize healthy eating over pleasure”) rated on a 4-point scale ranging from 1, *this does not apply to me*, to 4, *this applies to me* (Cronbach’s α = 0.82, McDonald's ω = 0.80). Finally, the Eating Disorders in Youth-Questionnaire (EDY-Q; [52]), adapted and validated for adults in Germany [53], examined ARFID symptoms through 14 questions encompassing food avoidance, selective eating, functional dysphagia, and issues related to underweight on a 7-point scale ranging from 0, *never true*, to 6, *always true* (Cronbach’s α = 0.66, McDonald's ω = 0.61). The total score is represented by the mean score of items 1 to 5 and 8 to 12, as per the convention of the instrument.

#### Help-seeking intentions

We measured help-seeking intentions with an item from the validated German version of the Stages of Change Questionnaire for Eating Disorders (SOCQ-ED; [54]). The SOCQ-ED is based on the transtheoretical model of change [55] and gauges a participant’s motivation to change specific ED symptoms and the overall intention to seek treatment for disordered eating, with a 7-point scale reflecting different intentional states (no need for therapy, precontemplation, contemplation, preparation, action, maintenance, termination; coded from 0 to 6). Because the overall item directly measured help-seeking intentions, we included it as the primary outcome variable in the present study.

#### Stigma-related perceptions of disordered eating in men

In the absence of a pre-existing (German) measure on stigma-related perceptions of EDs in men, we developed a set of seven items based on the lived experiences of men with EDs identified by Richardson and Paslakis [28]. Specifically, through discussion and drawing upon our clinical expertise, we formulated items that addressed perceived structural, public, and internalized aspects of stigmatization (e.g., “There are hardly any specialized treatment services for men with eating disorders.”, “Doctors take eating disorders less seriously in men than in women.”, “It is better for a man to not admit having an eating disorder”). The items were rated on a 4-point scale from 1, *completely disagree*, to 4, *completely agree*, and aggregated such that higher scores indicated higher perceived stigma. Table 1 shows the complete item list. The mean score had approximately acceptable internal consistency (Cronbach’s α = 0.69, McDonald’s ω = 0.71).

**Table 1:** Items and descriptive characteristics of the self-developed stigma-related perceptions of eating disorders in men measure

Item	*M*	*SD*
1. When men eat too little, are obese or exercise excessively, it is usually not due to an eating disorder	2.45	0.69
2. Eating disorders are women's diseases	1.48	0.69
3. It is better for a man to not admit having an eating disorder	1.33	0.66
4. Men with eating disorders have a harder time finding treatment than women	2.26	0.79
5. Doctors take eating disorders less seriously in men than in women	2.39	0.92
6. There are hardly any specialized treatment services for men with eating disorders	2.39	0.90
7. Eating disorders are less treatable for men than for women	1.82	0.66
Total score	2.02	0.45

*N* = 132. Items were rated on a 4-point scale from 1 (completely disagree) to 4 (completely agree) and aggregated such that higher scores indicated higher stigma-related perceptions of eating disorders in men

### Data aggregation and analysis

The disordered eating questionnaires were aggregated according to their convention. Variable values are reported as means (*M*) and standard deviations (*SD*). We screened for multivariate outliers across questionnaire scores using Mahalanobis distance with a criterion of *p* < 0.001. The internal consistency of the questionnaire scales was assessed using Cronbach’s α and McDonald's ω coefficients. The items on stigma-related perceptions of EDs in men were submitted to a single-factor confirmatory factor analysis (CFA) before aggregation. The CFA was conducted in R version 4.3.1 [56] using package lavaan version 0.6–17 [57] with maximum likelihood estimation and standardization of both observed and latent variable covariances. Model fit was assessed based on common fit indices, i.e., Comparative Fit Index (CFI), Root Mean Squared Error of Approximation (RMSEA), Standardized Root Mean Square Residual (SRMR), and Tucker–Lewis Index (TLI). In accordance with previous literature and recommendations [58, 59], we considered the model to fit the data well when CFI and TLI ≥ 0.95, RMSEA < 0.06 and SRMR < 0.08. For the main analyses we used Pearson correlations to examine bivariate associations. Moderator analyses, with disordered eating as the predictor, stigma-related perceptions of EDs in men as the moderator, and help-seeking intentions as the criterion, were performed using the PROCESS macro version 4.2 [60] for IBM SPSS 29 [61], with bootstrapped (5,000 samples) bias-corrected 95% confidence interval (CI) and heteroscedasticity correction (HC3). Predictor and moderator variables were mean-centered. Simple slopes were evaluated at the moderator mean (*M*) and *M* ± 1 *SD* and, more granular, using the Johnson-Neyman technique [62]. The significance level for all analyses was set at *p* ≤ 0.05. We used case-wise exclusion across analyses for missing data (1.9%). Plots for moderations were created using the ggplot2 package version 3.4.3 [63] in R version 4.3.1 [56] and an online visualization tool [64].

## Results

### Sample characteristics

Table 2 summarizes the sample sociodemographic characteristics, disordered eating symptoms, and help-seeking intentions. We did not detect multivariate outliers. Consistent with the selection of men without EDs, the overall symptom endorsement and help-seeking intentions remained low. The EDE-Q scores correlated positively with the MDDI and DOS, but not with the EDY-Q (Table 3). Age was negatively correlated with muscle dysmorphic symptoms (MDDI) but not with the other disordered eating scores. Stigma-related perceptions of EDs in men were pronounced in younger men and those who were single; help-seeking intentions did not vary by sociodemographic features (Table 4).

**Table 2:** Sample characteristics, disordered eating symptoms, and help-seeking intentions

Parameter	Total	Min	Max
n	132		
Age (years)	33.4 (12.4)	18.0	68.0
BMI (kg/m^2^)	26.2 (5.8)	17.1	52.3
*Gender*			
Male	131 (99.2%)		
Diverse	1 (0.8%)		
*Sexual orientation*			
Women	112 (84.8%)		
Men	16 (12.1%)		
Women and men	3 (2.3%)		
Other	1 (0.8%)		
*German language proficiency*			
First language	128 (97.0%)		
Fluent	4 (3.0%)		
*Educational attainment*			
Less than 12 years	22 (16.7%)		
12 years or more	110 (83.3%)		
*Migration background*			
Yes	14 (10.6%)		
No	118 (89.4%)		
*Marital status*			
Married	37 (28.0%)		
Single	91 (68.9%)		
Divorced	4 (3.0%)		
*Living situation*			
Alone	39 (29.5%)		
With others	93 (70.5%)		
EDE-Q global mean	1.34 (1.16)	0.00	4.73
DOS sum score	17.23 (4.66)	10	32
EDY-Q^a^ mean	1.21 (0.76)	0.00	4.20
MDDI sum score	26.47 (7.71)	13	53
Help-Seeking Intentions (SOCQ-ED)	0.49 (0.98)	0.00	6.00

Values are shown in n (%) or means and standard deviations (in brackets). Min = Minimum Value; Max = Maximum Value. EDE-Q = Eating Disorder Examination-Questionnaire; DOS = Duesseldorf Orthorexia Scale; EDY-Q = Eating Disorders in Youth-Questionnaire; MDDI = Muscle Dysmorphic Disorder Inventory; SOCQ-ED = Stages of Change Questionnaire for Eating Disorders

^a^*N* = 118 due to missing responses

**Table 3:** Bivariate variable correlations

Variable	*N*	*M*	*SD*	1	2	3	4	5	6	7
1. Age	132	33.36	12.39	1						
2. BMI	132	26.20	5.83	0.27**	1					
3. EDE-Q	132	1.34	1.16	0.07	0.44**	1				
4. DOS	132	17.23	4.66	− 0.07	0.14	0.52**	1			
5. EDY-Q^a^	118	1.21	0.76	− 0.03	− 0.14	0.13	− 0.03	1		
6. MDDI	132	26.47	7.71	− 0.24**	0.07	0.62**	0.56**	0.32**	1	
7. Stigma	132	2.02	0.45	− 0.19*	− 0.08	0.11	0.25**	0.09	0.37**	1

BMI = Body Mass Index; EDE-Q = Eating Disorder Examination-Questionnaire; DOS = Duesseldorf Orthorexia Scale; EDY-Q = Eating Disorders in Youth-Questionnaire; MDDI = Muscle Dysmorphic Disorder Inventory; Stigma = Stigma-related perceptions of eating disorders in men

^a^*N* = 118 due to missing responses

^*^p < 0.05. **p < 0.01

**Table 4:** Perceived stigma and help-seeking intentions by sociodemographic characteristics

Educational attainment	< 12 years (*n* = 22)	> 12 years (*n* = 110)	*t*	*p*	*d*
Stigma	1.94 (0.45)	2.03 (0.45)	− 0.95	0.352	− 0.22
SOCQ-ED	0.45 (0.86)	0.49 (1.00)	− 0.16	0.874	− 0.04

Marital status	Single (*n* = 91)	Married (*n* = 37)	*t*	*p*	*d*
Stigma	2.10 (0.43)	1.90 (0.40)	2.33	0.021	0.46
SOCQ-ED	0.49 (0.86)	0.24 (0.60)	1.89	0.062	0.32

Living situation	Alone (*n* = 39)	With others (*n* = 93)	*t*	*p*	*d*
Stigma	2.01 (0.47)	2.02 (0.44)	− 0.04	0.965	− 0.01
SOCQ-ED	0.44 (0.94)	0.51 (1.00)	− 0.37	0.711	− 0.07

Sexual orientation	Non- heterosexual (*n* = 19)	Heterosexual (*n* = 112)	*t*	*p*	*d*
Stigma	2.17 (0.46)	1.99 (0.44)	1.61	0.111	0.40
SOCQ-ED	0.63 (1.12)	0.46 (0.96)	0.69	0.493	0.17

Values show means and standard deviations (in parentheses). Stigma = Stigma-related perceptions of eating disorders in men; SOCQ-ED = Stages of Change Questionnaire for Eating Disorders

### Men’s ED stigma and help-seeking intentions

**Fig. 1:**
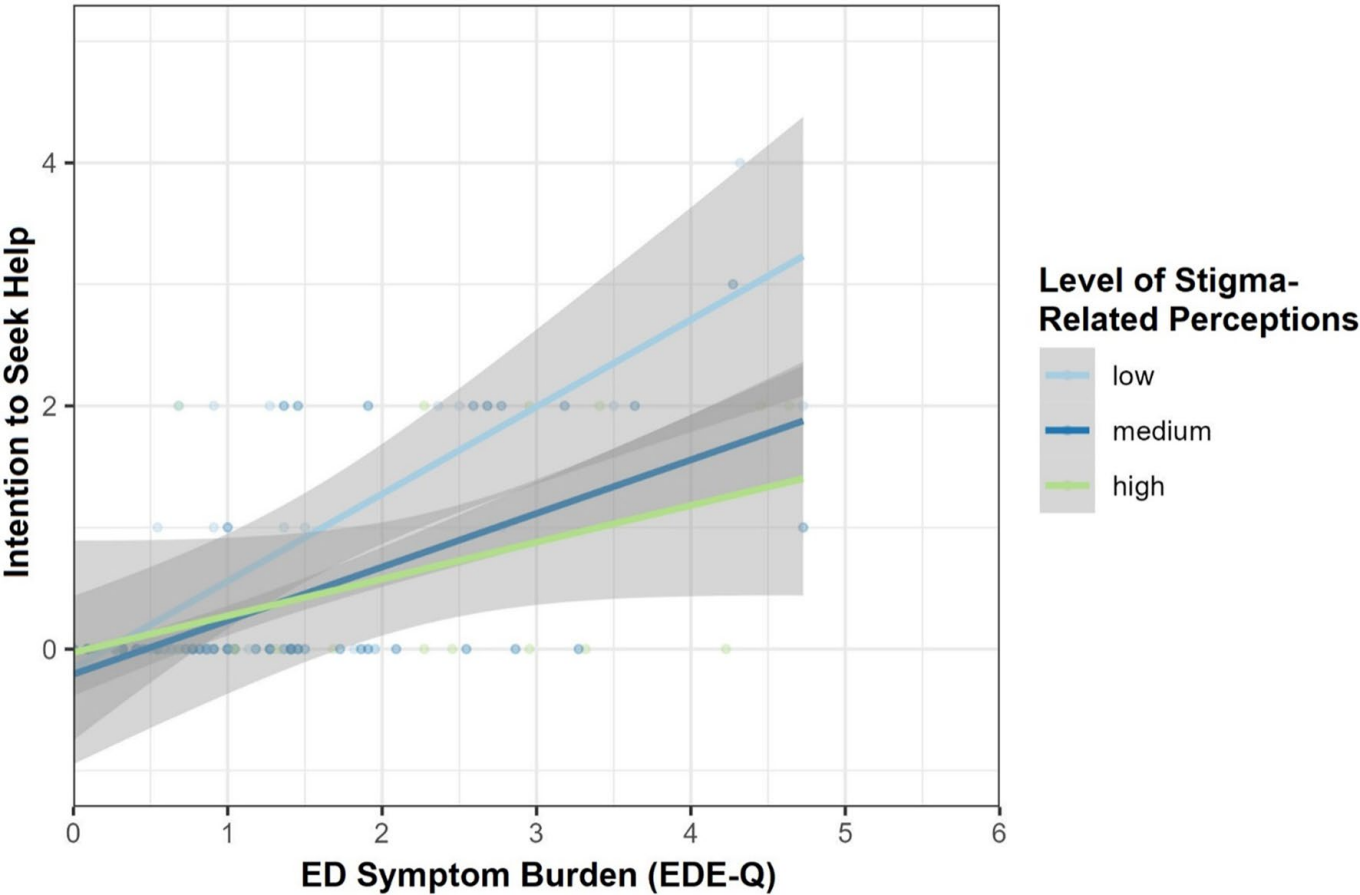
Moderating Effect of Stigma-Related Perceptions of Eating Disorders in Men on the Relationship Between ED Symptom Severity and Help-Seeking Intentions. ED = eating disorder. EDE-Q = Eating Disorder Examination-Questionnaire

**Fig. 2:**
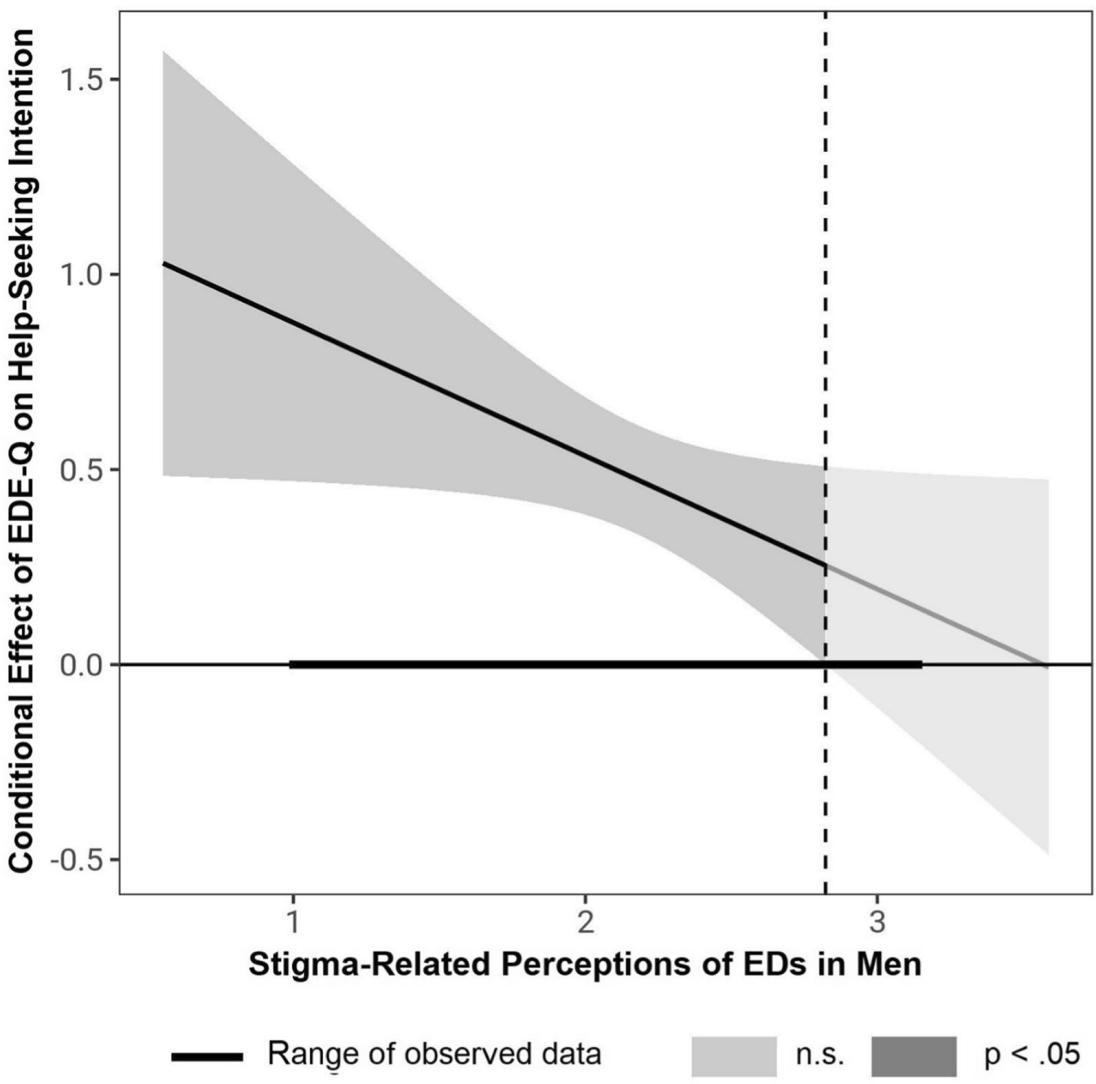
Johnson-Neyman Significance Regions for the Conditional Effects of Symptom Severity on Help-Seeking Intentions at Different Values of Stigma-Related Perceptions of Eating Disorders in Men. The effect is significant at stigma-related perceptions values below the dashed, vertical line (2.82 on a scale of 1 to 4). ED = eating disorder. EDE-Q = Eating Disorder Examination-Questionnaire

The CFA showed that a single latent factor fits the data well (CFI = 0.99, RMSEA = 0.02, SRMR = 0.05, TLI = 0.99; for full results, see Appendix). Because the items were designed to capture the full range of previously reported stigma-related experiences [28], we aggregated all items into a single mean score for further analyses to preserve the measure’s construct validity, despite individual low-loading items.^1^ The overall endorsement of stigma-related perceptions of EDs in men fell between slight disagreement and slight agreement, *M* = 2.02, *SD* = 0.45 (see Table 1). The moderator analysis with EDE-Q scores as predictor *X*, stigma-related perceptions as moderator *W*, and help-seeking intentions as criterion *Y*, was significant, *F*(3, 128) = 16.71, *p* < 0.001, *R*2 = 0.41. As expected, increased EDE-Q scores were associated with increased help-seeking intentions, *b*_X→Y_ = 0.53, *SE* = 0.08, *p* < 0.001, 95% CI [0.39; 0.68]. Stigma-related perceptions were not directly associated with help-seeking intentions, *b*_W→Y_ = -0.37, *SE* = 0.22, *p* = 0.09, 95% CI [− 0.78; 0.02]. However, stigma-related perceptions modulated the effect of symptom severity on help-seeking intentions, *b*_X*W→Y_ = − 0.34, *SE* = 0.17, *p* = 0.04, 95% CI [− 0.66; − 0.02], *R*^2^_change_ = 0.04. Consistent with our prediction, simple slope analysis (see Fig. 1) showed that the effect of symptom severity on help-seeking intentions decreased with increasing levels of stigma-related perceptions, *b*_−1SD_ = 0.68, *b*_M_ = 0.53, *b*_+1SD_ = 0.38, although the effect remained significant at the evaluated mean ± 1 *SD* levels, all *p*s < 0.001. However, the more granular Johnson-Neyman analysis showed that EDE-Q scores were only associated with help-seeking intentions until a stigma-related perceptions score of 2.82 (i.e., a tendency toward slight agreement), and became non-significant afterward, consistent with the idea that stigma-related perceptions are associated with reduced help-seeking for EDs in men (see Fig. 2). The results remained unaltered when including sociodemographic factors associated with reduced help-seeking such as low educational status, young age, and never-married status as covariates [65].

We further explored moderations by stigma-related perceptions for the effects of other disordered eating symptoms (see Table 5). The model with muscle dysmorphia (MDDI) scores as predictor was significant, *F*(3, 128) = 6.51, *p* < 0.001, *R*^2^ = 0.16. Higher MDDI scores and lower stigma-related perceptions predicted more help-seeking intentions, *b*_X→Y_ = 0.05, *SE* = 0.01, *p* < 0.001, 95% CI [0.03; 0.08] and *b*_W→Y_ = − 0.65, *SE* = 0.29, *p* = 0.03, 95% CI [− 1.22; − 0.13], respectively. However, stigma-related perceptions did not modulate the effect of the MDDI, *b*_X*W→Y_ = -0.01, *SE* = 0.02, *p* = 0.56, 95% CI [− 0.05; 0.04]. The model with orthorexic symptoms (DOS) as predictor was also significant, *F*(3, 128) = 3.38, *p* = 0.020, *R*^2^ = 0.13. More pronounced DOS scores predicted increased help-seeking intentions, *b*_X→Y_ = 0.07, *SE* = 0.02, *p* = 0.009, 95% CI [0.03; 0.12], but stigma-related perceptions had neither a direct influence nor did it modulate the effect of the DOS, *b*_W→Y_ = − 0.49, *SE* = 0.25, *p* = 0.05, 95% CI [− 1.00; -0.02] and *b*_X*W→Y_ = − 0.03, *SE* = 0.05, *p* = 0.57, 95% CI [− 0.13; 0.07], respectively. Finally, EDY-Q ARFID scores were unrelated to help-seeking intentions and were not modulated by stigma-related perceptions, *F*(3, 114) = 1.03, *p* = 0.382, *R*^2^ = 0.05. Thus, we did not obtain any evidence that stigma-related perceptions of EDs in men modulated the effects on help-seeking intentions of disordered eating symptoms besides those assessed by the EDE-Q.

**Table 5:** Moderation analyses for stigma-related perceptions

Relationship	Model statistics			Path statistics			
	*F*	*df1, df2*	*p*	*b*	*SE*	*p*	95% CI [*LL, UL*]
EDE-Q	16.71	3, 128	< 0.001**				
X → Y				0.53	0.08	< 0.001**	0.39; 0.68
W → Y				− 0.37	0.22	0.091	− 0.78; 0.02
X*W → Y				− 0.34	0.17	0.040*	− 0.66; − 0.02
MDDI	6.51	3, 128	< 0.001**				
X → Y				0.05	0.01	< 0.001**	0.03; 0.08
W → Y				− 0.65	0.29	0.026*	− 1.22; − 0.13
X*W → Y				− 0.01	0.02	0.561	− 0.05; 0.04
DOS	3.38	3, 128	0.020*				
X → Y				0.07	0.02	0.009*	0.03; 0.12
W → Y				− 0.49	0.25	0.053	− 1.00; − 0.02
X*W → Y				− 0.03	0.05	0.567	− 0.13; 0.07
EDY-Q	1.03	3, 114	0.382				
X → Y				0.02	0.12	0.844	− 0.23; 0.24
W → Y				− 0.50	0.30	0.119	− 1.13; 0.06
X*W → Y				− 0.18	0.40	0.681	− 0.97; 0.65

Values were computed with PROCESS version 4.2 model 1 using bootstrapping procedure with 5000 iterations, heteroscedasticity correction (HC3), and mean centering of predictor and moderator variables. X = respective symptom severity as independent variable; Y = intention to seek help as dependent variable; W = mean score of stigma-related perceptions of eating disorders in men items as moderator. EDE-Q = Eating Disorder Examination-Questionnaire; MDDI = Muscle Dysmorphic Disorder Inventory; DOS = Duesseldorf Orthorexia Scale; EDY-Q = Eating Disorders in Youth-Questionnaire; CI = confidence interval; LL = lower limit; UL = upper limit

*p < 0.05. **p < 0.01

## Discussion

Despite an increasing number of cases, men remain underrepresented in settings for the treatment of disordered eating. Previous studies found that men with disordered eating report more stigma towards seeking psychological help in general, which has been attributed to a feminized construction of EDs impeding help-seeking behavior [28, 29]. Here, we investigated whether stigma-related perceptions of EDs in men moderated the association between symptom severity and help-seeking intentions. Corroborating previous assumptions, we found that higher levels of disordered eating symptoms, as assessed by a standard measure of ED psychopathology (EDE-Q), predicted men’s intention to seek help and that the effect diminished with increasing levels of endorsed stigma-related perceptions of EDs in men. Specifically, disordered eating symptoms ceased to be associated with help-seeking intentions with even slight levels of stigma-related perceptions of EDs (2.82 on a scale of 1 to 4), which suggests that perceived stigmatization could possibly prevent men from seeking help for self-recognized symptoms.

Notably, the present findings provide initial evidence for the specificity of stigma-related barriers to help-seeking. Previous studies associated disordered eating in men with a broad range of symptoms, including muscularity-oriented, orthorexic, and avoidant/restrictive forms of eating behaviors [15–17]. We therefore assessed these behaviors, which mostly correlated positively with more traditional, “feminized” disordered eating symptoms (i.e., thinness, weight concern as assessed by EDE-Q). We also found that more pronounced muscle dysmorphia and orthorexic symptoms similarly predicted help-seeking intentions. ARFID symptoms were unrelated to help-seeking intentions and the association was also not modulated by stigma-related perceptions, although the low reliability of the measure found here and in other studies [53] limits interpretations. However, stigma only modulated the effect of the traditional, but not of the other disordered eating symptoms, for predicting help-seeking intentions, which directly supports that a “feminized” construction of EDs, and not of disordered eating per se, may impede access to care [29]. While the overall effects of a broad range of symptoms on help-seeking intentions highlight that men may benefit from more inclusive assessments in clinical practice, the potential specificity of the stigma-related moderation reiterates the importance of continuing efforts to tackle stigmatized perceptions of EDs as a “women’s disease” [66].

Although the CFA conducted showed that the different aspects assessed by the stigma-related perceptions item set could be summarized as a single construct, as the results were discussed previously, the supplementary exploratory factor analysis (EFA, see Appendix) suggested splitting the item set into two factors. This suggests that men’s stigma-related perceptions of ED treatment may be more specifically linked and therefore more relevant to help-seeking behavior than a single-item factor of more general gendered ED stereotypes. However, both factors represent men's perceptions of EDs in men compared to women, supporting the notion that it may yet be gendered perceptions and constructions of EDs which are a specific barrier to treatment for men. Furthermore, given the weak and unstable nature of factors with fewer than three items [67], further interpretation should be undertaken with considerable caution.

### Limitations

Despite the data supporting several of our assumptions and corroborating previous research, it is important to note some limitations. First, we included a general population sample and excluded individuals with a history of EDs to gauge effects on help-seeking intentions in the absence of prior ED-related help-seeking experiences. However, men with a diagnosed ED are potentially more affected by ED-related stigma and could show different levels of stigma-related perceptions. Indeed, the observed overall low endorsement levels of stigma-related perceptions and disordered eating symptoms may not be representative of a clinical sample. A full assessment of the association with stigma-related perceptions, therefore, requires replicating the present findings in men with diagnosed EDs. Second, we examined the effects of stigma cross-sectionally, which limits inferences concerning the causality of observed associations. Future studies should ideally include longitudinal data. Third, our study design, by using gender-specific wording and assessing gender at the beginning of the survey, may have contributed to an increased salience of gender among respondents and thus may have amplified the influence of stigma-related perceptions. We also advertised the survey as developing an online screening for EDs in men. Although transparent and anonymous, it cannot be ruled out that this may have deterred some people from participating. Relatedly, recruitment via social networks could lead to an overestimation or distortion due to snowballing effects, although we have minimized this risk by implementing a broader recruitment strategy. Although we screened for multivariate outliers and repeated participations, we did not embed attention check items into the survey, which may be considered a limitation in terms of data quality for data collected online. Fourth, we must recognize that stigma is a complex phenomenon. While we have focused here on the potential effects of a feminized construction of EDs, other facets such as weight stigma remain unexamined in this study. Future studies should therefore examine the multi-dimensionality of stigma as well as systematically assess potentially related and underlying constructs and processes, such as the precariousness of manhood or gender typing. Aggravatingly, there is a lack of consensus regarding terminology and theory within the field of stigma research [68], which should also be the focus of future efforts, as this could guide the development of more sophisticated measurement instruments and further advances in the field. For example, our items were based on a comprehensive systematic review of experiences of men with EDs [28], but future studies should aim to improve consistency while maintaining content validity. Fifth, we focused on one specific aspect of ED-related perceived stigma but did not assess its interplay with further stigma-related barriers to treatment (e.g., the likelihood of receiving a diagnosis or the general availability of specialized services; [69]). Help-seeking is a complex, multi-stepped process of evaluating one’s symptoms, gaining awareness of and accepting the problem, determining the cost and benefits of seeking help, and finally seeking and utilizing specialized services [70]. Thus, stigma may impede help-seeking intentions and behaviors in various yet unexplored ways. While using a single-item measure to assess help-seeking is efficient, it does not capture help-seeking behavior in its full complexity. Therefore, future studies need to consider utilizing more nuanced and multifaceted measures of help-seeking. Finally, complexity and precision could be added by assessing the effect of stigma through an intersectional lens, as male gender is only one of the various aspects of diversity that make up a person’s identity and thus may become subject to stigmatization.

## Conclusions

EDs are an often overlooked but increasingly relevant health problem in men. Perceived stigma towards men with EDs reduces the relation between “traditional” ED symptom severity and men’s intention to seek help, and thus potentially acts as a barrier to treatment for men. In this respect, the present study may be seen as an initial empirical indication of potential adverse effects of a feminized construction of EDs on help-seeking in men with disordered eating behavior, although the absence of this mechanism in women has yet to be demonstrated to confirm the assumed gender specificity. Understanding how stigma limits access to care may help to inform the development of stigma-reducing interventions on individual, societal, and structural levels [41]. It may ultimately help tear down the invisible walls towards adequate, timely, and appropriate treatment.

## Figures and Tables

**Fig. 3:**
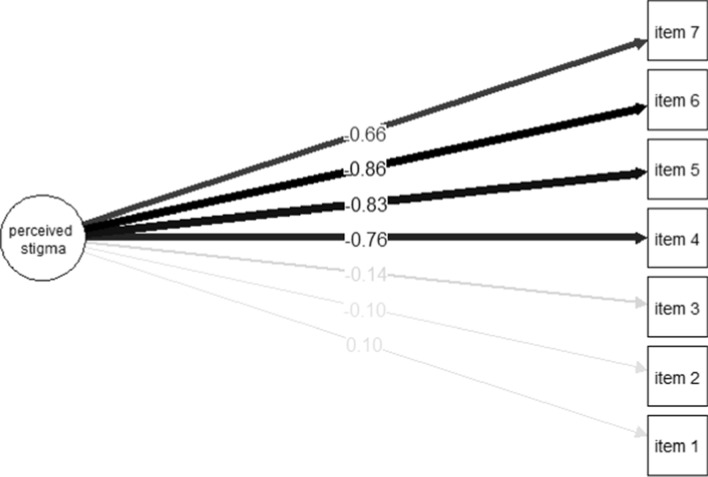
Latent variable model showing relationships between items and perceived stigma

**Fig. 4:**
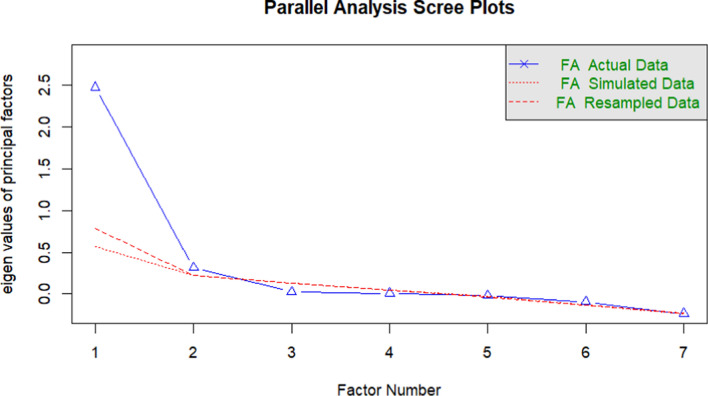
Horn’s parallel analysis

## Data Availability

The dataset and materials can be obtained from the corresponding author upon request.

## Appendix

### Confirmatory factor analysis: full results

The items on stigma-related perceptions of EDs in men were submitted to a single-factor confirmatory factor analysis (CFA) before aggregation. The CFA was conducted in R version 4.3.1 [56] using package lavaan version 0.6–17 [57] with maximum likelihood estimation and standardization of both observed and latent variable covariances. Model fit was assessed based on common fit indices, i.e., Comparative Fit Index (CFI), Root Mean Squared Error of Approximation (RMSEA), Standardized Root Mean Square Residual (SRMR), and Tucker–Lewis Index (TLI). In accordance with previous literature and recommendations [58, 59], we considered the model to fit the data well when CFI and TLI ≥ 0.95, RMSEA < 0.06 and SRMR < 0.08. The conducted CFA showed that a single latent factor model fits the data well (CFI = 0.99, RMSEA = 0.02, SRMR = 0.05, TLI = 0.99). Standardized parameter estimates for the model are presented in Table

**Table 6:** Standardized factor loadings for latent constructs from confirmatory factor analysis

Item	Standardized factor loading	*SE*	*z*	*p*
1	0.10	0.09	1.05	0.295
2	− 0.10	0.09	− 1.07	0.286
3	− 0.14	0.09	− 1.48	0.140
4	− 0.76	0.08	− 9.71	< 0.001
5	− 0.83	0.08	− 10.99	< 0.001
6	− 0.86	0.07	− 11.64	< 0.001
7	− 0.66	0.08	− 8.10	< 0.001

The confirmatory factor analysis was conducted using the Maximum Likelihood estimation with the NLMINB optimization method and standardization of both observed and latent variable covariances. CFI = 0.99, RMSEA = 0.02, SRMR = 0.05, TLI = 0.99

^6^ and Fig. 3. Overall, the results support the validity of the measurement model and the hypothesized single-factor structure.

Despite the adequate fit, the internal consistency of the items was suboptimal (Cronbach’s α = 0.69, McDonald’s ω = 0.71). We therefore submitted the items of our stigma-related perceptions measure to an additional exploratory factor analysis (EFA) and re-analyzed our data based on the suggested splitting of items.

#### Exploratory factor analysis

The EFA was performed using the maximum likelihood extraction method and orthogonal rotation based on Varimax procedure. The analysis was conducted in R version 4.3.1 [56] using package psych 2.4.3 [71]. The Kaiser–Meyer–Olkin measure of sampling adequacy (KMO) and Bartlett’s test of sphericity were used to ensure the conductibility of the EFA. The KMO resulted in a value of 0.8, which is higher than the minimum value of 0.5 [72], indicating that a sufficient amount of variance of variables could be explained by underlying factors. Additionally, Bartlett’s test of sphericity reached statistical significance (χ2 = 252.91, *p* < 0.001), indicating that the data were suitable for factor analysis. Horn’s parallel analysis (see Fig. 4) suggested extracting two factors. After extraction, items 2 and 3 were removed due to below-threshold communalities of lower than 0.30. We further removed items with factor loadings lower than 0.40, which is a common criterium for items to be clinically meaningful [73]. The remaining items were then assigned to the factor in which they had the highest loading. Thus, the EFA suggested averaging items 4 to 7 as one factor and maintaining item 1 as a second factor (see Table

**Table 7:** Factor structure of stigma-related perceptions of eating disorders in men measure by exploratory factor analysis

Item	Factor loading		Communality
	Factor 1	Factor 2	
1	− 0.17	**0.57**	0.35
2	0.08	0.15	0.03^a^
3	0.10	0.35	0.13^a^
4	**0.78**	− 0.06	0.60
5	**0.82**	0.13	0.68
6	**0.86**	0.07	0.74
7	**0.64**	0.27	0.48

The extraction method was maximum likelihood extraction with orthogonal varimax rotation method. Bold type indicates the respective loadings of the items that were crucial for the assignment to the respective factor

^a^Item removed due to below-threshold communality and clinical meaningfulness

7).

#### Re-analysis of the data based on the two-factor solution

We re-analyzed the data using the mean score of items 4 to 7 (moderator *W*) and item 1 responses (moderator *Z*) as two independent moderators in PROCESS version 4.2, with bootstrapped (5000 samples) bias-corrected 95% confidence interval (CI), heteroscedasticity correction (HC3), and mean centering of predictor and moderator variables. As in the primary analysis, intention to seek help served as criterion *Y*, and the symptom severity measures as independent predictors *X*.

The association between the EDE-Q and help-seeking was primarily moderated by the score of items 4 to 7 (i.e., stigma-related perceptions for ED treatment), with item 1 (generally perceiving EDs as gender-stereotyped) having a reduced but non-significant effect. As in the primary analysis, no other moderations were detected (see Table

**Table 8:** Moderation analyses with two independent stigma-related perceptions of eating disorders in men categories

Relationship	Model statistics			Path statistics			
	*F*	*df1, df2*	*p*	*b*	*SE*	*p*	95% CI [*LL, UL*]
EDE-Q	9.93	5, 126	< 0.001**				
X → Y				0.53	0.08	< 0.001**	0.38, 0.68
W → Y				− 0.21	0.12	0.110	− 0.45, 0.03
Z → Y				− 0.20	0.10	0.038*	− 0.39, 0.01
X*W → Y				− 0.20	0.10	0.049*	− 0.39, 0.01
X*Z → Y				− 0.14	0.11	0.207	− 0.34, 0.11
MDDI	3.99	5, 126	0.002**				
X → Y				0.05	0.01	< 0.001**	0.03, 0.08
W → Y				− 0.39	0.19	0.041*	− 0.78, − 0.05
Z → Y				− 0.15	0.11	0.158	− 0.37, 0.06
X*W → Y				− 0.01	0.01	0.539	− 0.03, 0.02
X*Z → Y				− 0.01	0.01	0.389	− 0.04, 0.02
DOS	1.90	5, 126	0.098				
X → Y				0.07	0.02	0.006**	0.03, 0.12
W → Y				− 0.26	0.16	0.106	− 0.58, 0.04
Z → Y				− 0.18	0.11	0.112	− 0.41, 0.03
X*W → Y				− 0.03	0.03	0.345	− 0.09, 0.04
X*Z → Y				− 0.01	0.03	0.688	− 0.07, 0.04
EDY-Q	0.74	5, 112	0.595				
X → Y				0.02	0.13	0.886	− 0.26, 0.26
W → Y				− 0.30	0.19	0.149	− 0.70, 0.04
Z → Y				− 0.21	0.14	0.177	− 0.48, 0.08
X*W → Y				− 0.09	0.28	0.781	− 0.67, 0.41
X*Z → Y				0.00	0.21	0.992	− 0.39, 0.45

Values were computed with PROCESS version 4.2 model 2 using bootstrapping procedure with 5000 iterations, heteroscedasticity correction (HC3), and mean centering of predictor and moderator variables. X = respective symptom severity as independent variable; Y = intention to seek help as dependent variable; W = mean score of stigma-related perceptions of eating disorders in men items 4 to 7 as moderator; Z = stigma-related perceptions of eating disorders in men item 1 as moderator; EDE-Q = Eating Disorder Examination-Questionnaire; MDDI = Muscle Dysmorphic Disorder Inventory; DOS = Duesseldorf Orthorexia Scale; EDY-Q = Eating Disorders in Youth-Questionnaire; CI = confidence interval; LL = lower limit; UL = upper limit

**p* < 0.05. ***p* < 0.01

^8^). The pattern of conditional effects also remained comparable when only including the score of items 4 to 7 as moderator, without item 1. This pattern could suggest a more prominent impact of stigma-related perceptions that specifically concern ED treatment on help-seeking intentions compared to general ED-related stereotypes. However, given that factors with less than three items are generally weak and unstable [67], and that a single-factor solution, as indicated by the reported CFA, fits the data well, we caution against further interpretation and opted to aggregate the items on perceived stigma into a single mean score for further analyses.

## Abbreviations

ARFID: Avoidant/restrictive eating disorder
CFA: Confirmatory factor analysis
CFI: Comparative fit index
DOS: Duesseldorf orthorexia scale
ED: Eating disorder
EDE-Q: Eating disorder examination-questionnaire
EDY-Q: Eating disorders in youth-questionnaire
EFA: Exploratory factor analysis
KMO: Kaiser–Meyer–Olkin measure of sampling adequacy
M: Mean
Max: Maximum value
MDDI: Muscle dysmorphic disorder inventory
Min: Minimum value
RMSEA: Root mean squared error of approximation
SD: Standard deviation
SOCQ-ED: Stages of change questionnaire for eating disorders
SRMR: Standardized root mean square residual
TLI: Tucker–Lewis index

## Footnotes

1 Despite the CFA showing adequate fit, the internal consistency of the items was suboptimal (Cronbach’s α = .69, McDonald's ω = .71). We therefore re-analyzed our data based on the splitting of items suggested by an exploratory factor analysis (EFA; see Appendix A). This re-analysis more specifically linked a four-item factor of stigma-related perceptions of ED treatment to help-seeking behavior rather than a single-item factor of more general gendered ED stereotypes. However, because the overall results and conclusions remained comparable when splitting the items into two factors or when only including the four-item factor, and due to the difficulty of interpreting single-item factors, we adhered to reporting the intended analyses using the overall mean.
